# Histological Characteristics of Myxoid (Metaplastic) Meningioma in a 44-Year-Old Woman

**DOI:** 10.1155/2019/9207632

**Published:** 2019-12-06

**Authors:** Akiko Marutani, Ryou Nakano, Noriyuki Nishi, Tomonori Yamada

**Affiliations:** ^1^Department of Neurosurgery, National Hospital Organization, Osaka Minami Medical Center, Osaka, Japan; ^2^Oshima Medical Association Hospital, Kagoshima, Japan

## Abstract

Myxoid (metaplastic) meningioma is the rarest WHO grade 1 meningioma, and its histological characteristics are useful in diagnostics. We present the case report of a myxoid (metaplastic) meningioma in a 44-year-old woman to highlight the important histological features and observations that are critical for making an accurate diagnosis. We report a rare myxoid meningioma using magnetic resonance imaging (MRI) images and its histopathological features.

## 1. Introduction

Meningiomas are tumors that develop from arachnoid cells; approximately 15 histological subtypes with various characteristics are known. Myxoid meningioma is an extremely rare subtype of WHO grade I benign meningioma; only eight cases have been reported [1]. Here, we report a case of myxoid (metaplastic) meningioma using MRI images and its histopathological features.

## 2. Case Report

**Figure 1:**
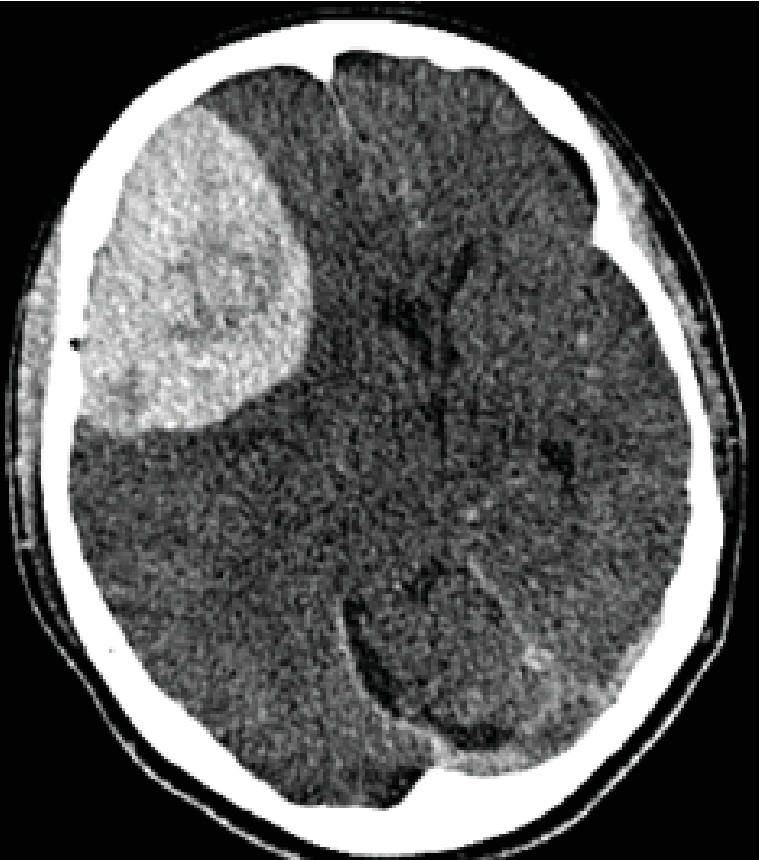
A head computed tomography (CT) scan showed a 90 × 90 mm mass in the right frontal lobe.

**Figure 2:**
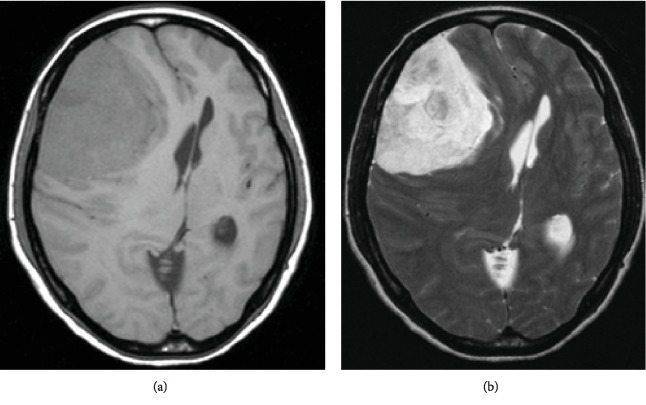
(a) Head magnetic resonance imaging (MRI) revealed a mass presenting homogeneous hypointense signals on T1 images. (b) At the same site, hyperintense signals with a clear border on T2 images.

**Figure 3:**
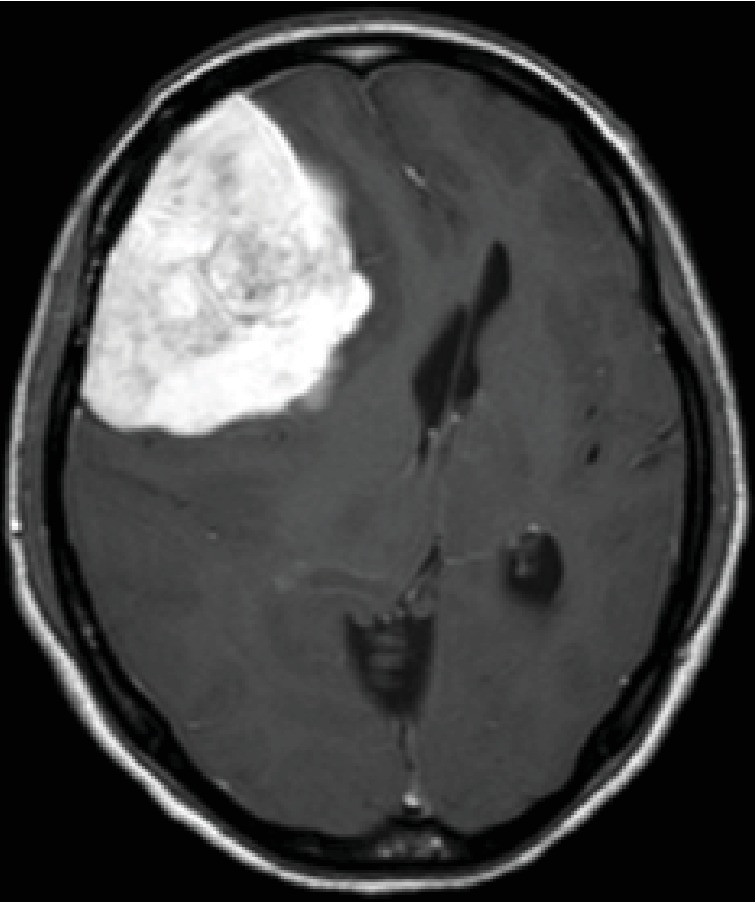
Gadolinium- (Gd-) based contrast-enhanced MRI significantly enhanced the signals.

A 44-year-old woman presented with a headache and visual impairment in August 2017 and visited a local doctor one month later. A head computed tomography (CT) scan showed a 90 × 90 mm mass in the right frontal lobe (Figure 1), and she was referred to our hospital. Head MRI revealed a mass presenting homogeneous hypointense signals on T1 images (Figure 2(a)) and hyperintense signals with a clear border on T2 images (Figure 2(b)) at the same site. Gadolinium- (Gd-) based contrast-enhanced MRI significantly enhanced the signals (Figure 3). On cerebral angiography, the mass had a sunburst appearance and refluxed from the middle meningeal artery (MMA). Based on these results, we diagnosed the mass a convexity meningioma and performed a craniotomy and tumorectomy.

**Figure 4:**
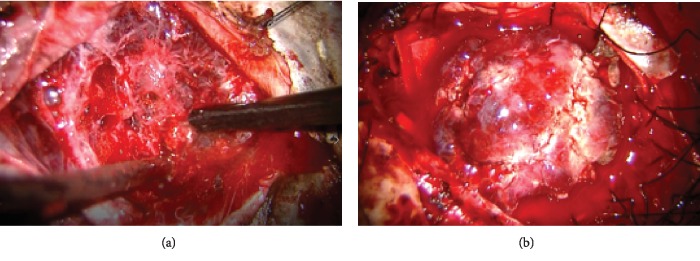
(a) Intraoperative findings showed that the tumor was hemorrhagic, dark red, and relatively soft and was partially accompanied by fibrous tissue. (b) After the MMA was treated, internal decompression was performed with Sonopet (Stryker Japan KK) to achieve total resection of the tumor, including removal of the infiltrated dura (Simpson grade I).

Intraoperative findings showed that the tumor was hemorrhagic, dark red, and relatively soft and was partially accompanied by fibrous tissue (Figure 4(a)). After the MMA was treated, internal decompression was performed with Sonopet (Stryker Japan KK) to achieve total resection of the tumor, including removal of the infiltrated dura (Simpson grade I) (Figure 4(b)).

**Figure 5:**
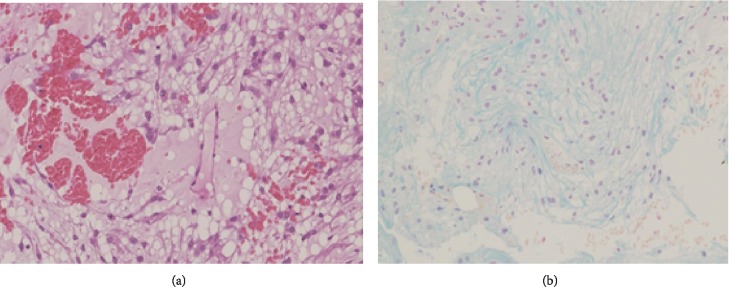
(a) Histopathological analysis showed that fibrous connective tissue containing numerous blood vessels was subfractioned into leaflet-like portions. (b) The areas inside the leaflets were stained with Alcian blue and contained a mucoid matrix.

Histopathological analysis showed that fibrous connective tissue containing numerous blood vessels was subfractioned into leaflet-like portions (Figure 5(a)). The areas inside the leaflets were stained with Alcian blue and contained a mucoid matrix (Figure 5(b)). Immunostaining revealed that the tumor was positive for vimentin and epithelial membrane antigen (EMA) and negative for glial fibrillary acidic protein (GFAP). A small percentage (4.4%) of tumor cells were positive for Ki-67, and there was no finding of malignancy. Based on the above findings, we diagnosed the tumor as myxoid meningioma.

After surgery, the neurologic deficits resolved and the patient's progress was favorable. The patient was discharged two weeks after surgery and was able to walk independently upon discharge. Two years after surgery, recurrence had not been observed.

Written informed consent was obtained from the patient for publication of this case report. A copy of the written consent is available for review upon request.

## 3. Discussion

Metaplastic meningioma is a subtype of tumor in which a metaplasia of cells into stromal cells occurs locally to form bone, cartilage, lipids, and mucus. It includes osseous, cartilaginous, lipomatous, myxoid, xanthomatous, and melanin meningiomas [2]. Till now, most of reports of the metaplastic meningioma were osseous, lipomatous, xanthomatous, and smooth muscle meningiomas [3].

Myxoid meningioma is a rare type of metaplastic meningioma; pathological and immunohistological diagnoses are important for definitive diagnosis and discrimination from other histological subtypes of meningioma and mucoid tumors (subtypes include osteosarcoma, chondrosarcoma, liposarcoma, chordoma, and xanthoma) [1]. In addition to whorl formation, psammoma body, and syncytial formation that are histopathological characteristics of meningioma, myxoid meningioma is characterized by an abundant mucoid matrix in the cytoplasm and large amounts of acidic mucopolysaccharides that stain with Alcian blue [4]. Our case is stained with Alcian blue and contained a mucoid matrix.

Grade II chordoid meningioma is an additional histological subtype of meningioma that commonly requires discrimination. Compared with myxoid meningioma, chordoid meningioma shows a higher grade of dyskaryosis and causes a reticular arrangement with less cytoplasmic vacuolation. In addition, lymphocytes are not observed in myxoid meningioma, while Castleman disease, which causes chordoid meningioma, is characterized by abundant lymphocytes [5].

Myxoid meningioma shows positive immunohistological reactivity for vimentin and EMA and negative reactivity for cytokeratin, HMB45, S-100, actin, desmin, CD117, neurofilaments, and GFAP [6]. A few metaplastic tumors showing positive immunoreactivity for smooth muscle action (SMA) suggest muscular differentiation [7].

We consider that the hemorrhagic, dark red characteristics are due to abundant vascularization. MRI showed significantly hyperintense signals on T2 images. The contrast was successfully enhanced using a Gd-based contrast agent due to the histologically abundant mucoid matrix and vascularization. Our histopathological analysis showed fibrous connective tissue containing numerous blood vessels. In our case, MRI showed very hyperdense signals on T2 images and enhanced Gd-based contrast.

Ki-67 (MIB-1) is used to examine tumor proliferative activity. After nuclear immunostaining, calculating the percentage of cells positive for Ki-67 enables prediction of tumor malignancy, prognosis, and recurrence [8]. In the present case, we were able to perform a total resection with no finding of malignancy. However, cases with a high recurrence, findings of malignancy, and a high score of Ki-67 have been reported; therefore, careful follow-up is necessary.

## 4. Conclusion

We report a rare case of myxoid (metaplastic) meningioma using MRI images and its histopathological features. However, its biological behavior is poorly understood because of the limited number of reported cases. This is a matter that demands full investigation for diagnosis.
